# Case in which percutaneous fibrin glue injection was useful for refractory urinary fistula following robot‐assisted partial nephrectomy

**DOI:** 10.1002/iju5.12724

**Published:** 2024-04-04

**Authors:** Nao Yukimatsu, Takeshi Yamasaki, Keiko Iguchi, Taiyo Otoshi, Minoru Kato, Junji Uchida

**Affiliations:** ^1^ Department of Urology, Graduate School of Medicine Osaka Metropolitan University Osaka Japan

**Keywords:** fibrin glue, robot‐assisted partial nephrectomy, urinary fistula

## Abstract

**Introduction:**

Urinary fistula is a rare complication following robot‐assisted partial nephrectomy. For cases refractory to conservative treatment, only ureteral stent placement and percutaneous drainage are the established treatment alternatives.

**Case presentation:**

A 44‐year‐old man presented with urinary fistula 3 weeks after robot‐assisted partial nephrectomy for right renal cell carcinoma. Follow‐up observations were conducted for 2 weeks; however, no improvements were observed. Additionally, the patient did not improve following percutaneous drainage and ureteral stent insertion. Subsequently, the patient received percutaneous injections of fibrin glue, with the urinary fistula showing significant improvements on the following day.

**Conclusion:**

Our findings indicated that percutaneous fibrin glue injection can effectively treat refractory urinary fistula following partial nephrectomy.

**Keynote message**

We report a case in which percutaneous fibrin glue injection was effective for a refractory urinary fistula following RAPN. Percutaneous fibrin glue injection may be an effective treatment for refractory urinary fistulas that do not improve with ureteral stent placement or percutaneous drainage.

**Abbreviations & Acronyms**
PN: partial nephrectomy
RAPN: robot‐assisted partial nephrectomy
RCC: renal cell carcinoma
RN: radical nephrectomy

## Introduction

PN is the standard treatment for patients with localized T1 RCC.^1^ Compared with RN, PN has superior functional outcomes and comparable oncological outcomes.^2^ However, urinary fistulas following RAPN have been reported to occur in 0.2–1.5% of cases.^3^ Urinary fistulas can be expected to spontaneously improve; therefore, conservative treatment is usually provided, as appropriate. However, for cases refractory to conservative treatment, there remain no established treatment alternatives other than ureteral stent placement and percutaneous drainage.^4^ We report a case in which percutaneous injection of fibrin glue effectively improved refractory urinary fistula following RAPN.

## Case presentation

**Fig. 1:**
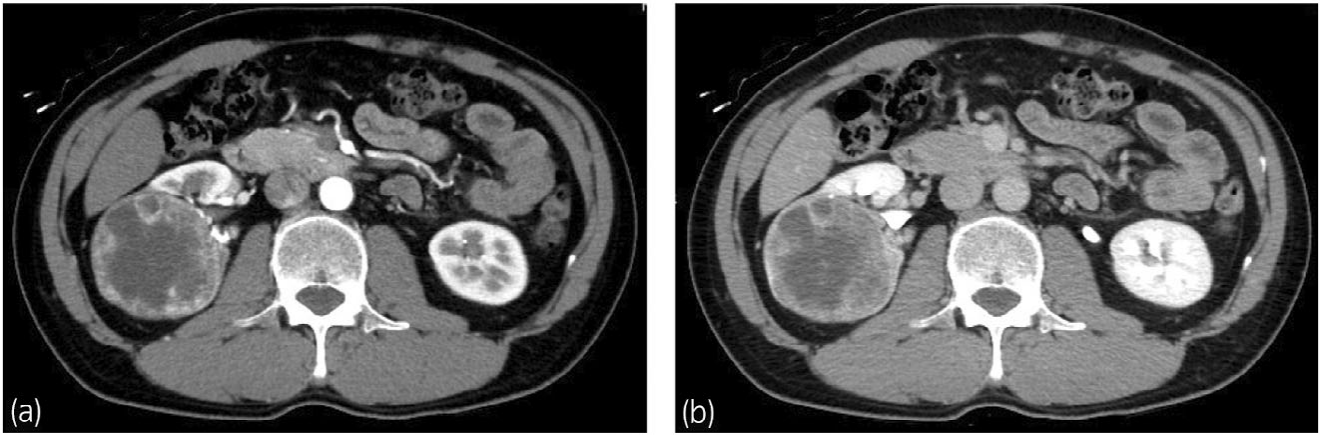
Preoperative computed tomography. A 67‐mm tumor with contrast enhancement was observed in the middle of the right kidney. The tumor is extensively proximal to the urinary tract. (a) Coronal section, arterial phase, (b) Coronal section, excretory phase.

**Fig. 2:**
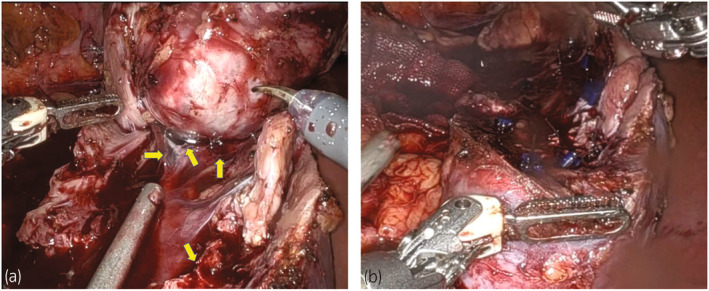
Intraoperative image. (a) Urinary tract opened in multiple places during tumor resection (arrows), (b) Following completion of resection bed suturing.

A 44‐year‐old man was referred to our department for treatment of a 67‐mm right renal mass. Based on imaging tests, the patient was diagnosed with a nonmetastatic clear cell RCC localized to the middle part of the right kidney with contrast enhancement; accordingly, RAPN was selected as the treatment (Fig. 1). RAPN was performed with the patient in a nephrectomy position using transabdominal approach. The renal pelvis was expected to open during tumor resection; therefore, a single‐J catheter was placed. Subsequently, physiological saline containing indigo carmine was perfused during tumor resection. The main trunk of the right renal artery was blocked during tumor resection. The urinary tract, which was opened at multiple sites during tumor resection, was repaired with continuous or interrupted sutures using 3‐0 MONOVRYL™ (ETHICON, New Brunswick, NJ, USA) (Fig. 2). Furthermore, a sheet of biological tissue adhesive, TachoSil^®^ (CSL Behring, King of Prussia, PA, USA), was applied to the resection bed to complete the procedure. The surgery, console, and ischemia durations were 250, 135, and 35 min, respectively; further, the blood loss amount was 600 mL. The pathological diagnosis was clear cell RCC, pT1b, Grade 2, with negative resection margins. Biochemical tests of drainage fluid on the third postoperative day (Cre 1.21 mg/dL, K 4.4 mmol/L) revealed no findings suggestive of a urinary fistula; accordingly, the drain was removed. The clinical course progressed without any significant changes, and the patient was discharged on the seventh postoperative day.

**Fig. 3:**
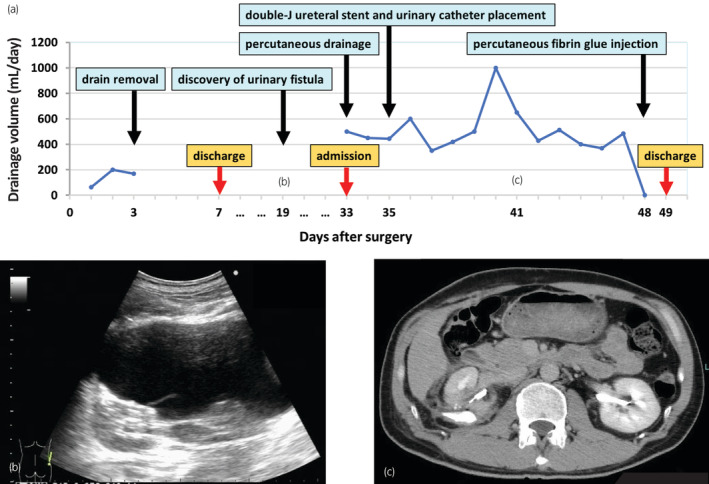
Clinical course of the case. (a) Time‐course change in therapies and drainage volume, (b) Right kidney ultrasound image on the 19th postoperative day. A hypoechoic image suggestive of urinary fistula is observed around the kidney, (c) Computed tomography on the 41th postoperative day. Leakage of contrast medium is observed around the right kidney (coronal section, excretory phase).

**Fig. 4:**
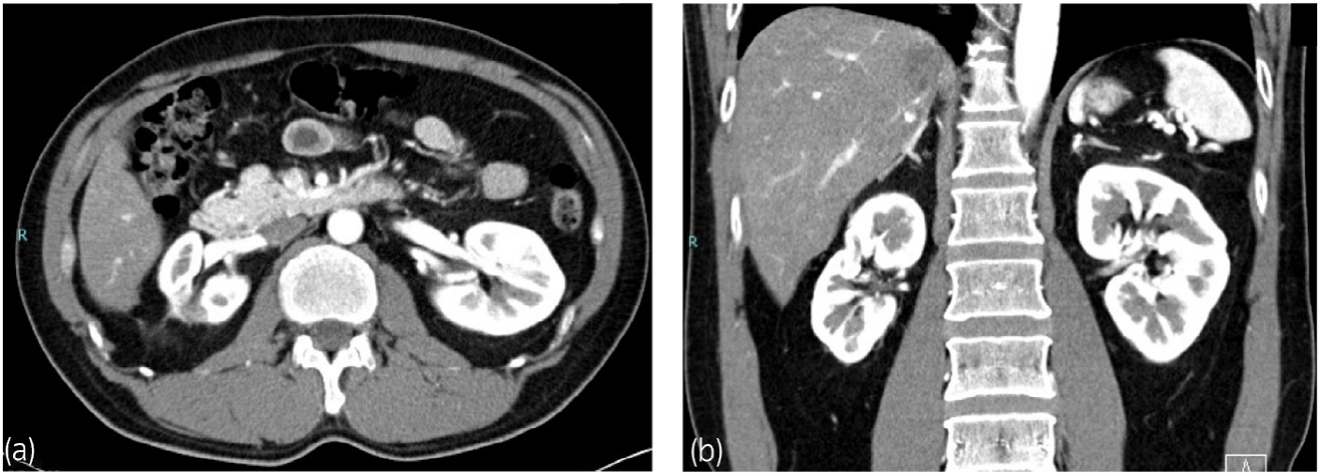
A computed tomography image obtained at two and a half postoperative years. There are no findings suggestive of a urinary fistula or RCC. (a) Coronal section, cortex phase, (b) Axial section, cortex phase.

On the 19th postoperative day, a renal ultrasound test conducted at the first postoperative outpatient visit revealed a 16 × 7 cm hypoechoic image around the right kidney, which was suggestive of a urinary fistula. Accordingly, the patient underwent follow‐up observations without any subjective symptoms or signs of infection. On the 33rd postoperative day, renal ultrasound tests revealed an increasing tendency for the hypoechoic image, and the patient was admitted to the hospital for treatment. On the day of admission, percutaneous drainage was performed (600 mL of urine collected), and a pig tail catheter was placed. On the third day of hospital stay, the drainage volume remained at approximately 400 mL/day with no improvement; moreover, double‐J ureteral stent and a urinary catheter were placed under spinal anesthesia. Even after placement of a double‐J ureteral stent, the drainage volume remained at approximately 500 mL/day. On the 15th day of hospital stay, a guide wire was inserted through the pig tail catheter; subsequently, a 12 Fr Nelaton catheter was placed around the kidney. Next, fibrin (solution A) and thrombin (solution B) from the fibrin glue Bolheal^®^ (Kaketsuken, Kumamoto, Japan) were diluted twofold with physiological saline, with 5 mL of each preparation being injected. This was followed by replacement with a 12 Fr nephrostomy balloon catheter. On the next day, there was no drainage from the nephrostomy balloon catheter; moreover, renal ultrasound tests did not show any hypoechoic images where a urinary fistula around the kidney was suspected. Accordingly, the nephrostomy balloon catheter was removed. There were no significant changes on the second day after fibrin glue injection, and the patient was discharged from the hospital (Fig. 3). The double‐J ureteral stent was removed 1.5 months after fibrin glue injection. There has been no recurrence of the urinary fistula or RCC (Fig. 4).

## Discussion

Urinary fistula is a complication following PN; however, its incidence has been decreasing with advances in research evidence and surgical techniques. Potretzke *et al*. reported that incidence of urinary fistula was 0.8% among 1791 cases of RAPN.^5^ Urinary fistulas are usually expected to spontaneously improve with follow‐up observations.^6^ However, if no improvements are observed, ureteral stent placement and percutaneous drainage can be attempted.^4^, ^5^ Other subsequent alternative treatment methods include percutaneous fibrin glue injection,^7^, ^8^, ^9^ percutaneous cyanoacrylate injection,^10^ transurethral fibrin glue,^11^ transurethral cyanoacrylate injection,^12^ selective renal artery embolization,^6^, ^13^ and desmopressin administration^14^; however, none of them are yet to be scientifically established.

Fibrin glue is a topical bioadhesive that utilizes a coagulation reaction by mixing plasma component‐derived fibrinogen (solution A) and thrombin (solution B); further, it is highly safe since it utilizes normal biological reactions.^15^ It is not only used to treat the renal pelvis and calyx, but also intestinal and vesicovaginal fistulas.^16^, ^17^ There have been three previous case reports of percutaneous injections of fibrin glue into refractory urinary fistulas following PN,^7^, ^8^, ^9^ with all cases showing improvement of the urinary fistula within a few days after fibrin glue administration. In our case, the urinary fistula disappeared the next day after injection of fibrin glue. This procedure can be conducted without anesthesia or by using only local anesthesia; therefore, it involves minimal invasiveness. Moreover, they have been no reports of complications such as urinary tract obstruction. Taken together, percutaneous injection of fibrin glue is a minimally invasive and highly effective treatment for refractory urinary fistulas.

Double‐layer rennorraphy using renal parenchymal sutures has been shown to involve a lower incidence of urinary fistula than single‐layer outer cortical rennorraphy with only a resection bed.^18^ Currently, in cases in which the urinary tract is expected to be largely opened by tumor resection, ureteral stents are not placed; however, double‐layer rennorraphy is performed using renal parenchymal sutures to prevent urinary fistulas.

## Conclusion

This article describes a case in which percutaneous fibrin glue injection was effective for refractory urinary fistula following RAPN. Our findings indicate that percutaneous fibrin glue injection could be an effective treatment for refractory urinary fistula following PN.

## Author contributions

Nao Yukimatsu: Conceptualization; data curation; investigation; methodology; validation; writing – original draft. Takeshi Yamasaki: Conceptualization; investigation; methodology; project administration; validation. Keiko Iguchi: Supervision. Taiyo Otoshi: Supervision. Minoru Kato: Supervision. Junji Uchida: Writing – review and editing.

## Conflict of interest

The authors declare no conflict of interest.

## Approval of the research protocol by an Institutional Reviewer Board

Not applicable.

## Informed consent

Not applicable.

## Registry and the Registration No. of the study/trial

Not applicable.
